# Effects of Online Single Pulse Transcranial Magnetic Stimulation on Prefrontal and Parietal Cortices in Deceptive Processing: A Preliminary Study

**DOI:** 10.3389/fnhum.2022.883337

**Published:** 2022-06-20

**Authors:** Bruce Luber, Lysianne Beynel, Timothy Spellman, Hannah Gura, Markus Ploesser, Kate Termini, Sarah H. Lisanby

**Affiliations:** ^1^Noninvasive Neuromodulation Unit, Experimental Therapeutics and Pathophysiology Branch, National Institute of Mental Health, Bethesda, MD, United States; ^2^Department of Neuroscience, University of Connecticut School of Medicine, Farmington, CT, United States; ^3^Department of Psychiatry and Neurosciences, University of California, Riverside, Riverside, CA, United States; ^4^Forensic Psychiatry, Department of Psychiatry, Faculty of Medicine, The University of British Columbia, Vancouver, BC, Canada; ^5^Clinical and Forensic Psychology, Fifth Avenue Forensics, New York, NY, United States

**Keywords:** TMS, deception, parietal cortex, fronto-parietal network, guilty knowledge task (GKT)

## Abstract

Transcranial magnetic stimulation (TMS) was used to test the functional role of parietal and prefrontal cortical regions activated during a playing card Guilty Knowledge Task (GKT). Single-pulse TMS was applied to 15 healthy volunteers at each of three target sites: left and right dorsolateral prefrontal cortex and midline parietal cortex. TMS pulses were applied at each of five latencies (from 0 to 480 ms) after the onset of a card stimulus. TMS applied to the parietal cortex exerted a latency-specific increase in inverse efficiency score and in reaction time when subjects were instructed to lie relative to when asked to respond with the truth, and this effect was specific to when TMS was applied at 240 ms after stimulus onset. No effects of TMS were detected at left or right DLPFC sites. This manipulation with TMS of performance in a deception task appears to support a critical role for the parietal cortex in intentional false responding, particularly in stimulus selection processes needed to execute a deceptive response in the context of a GKT. However, this interpretation is only preliminary, as further experiments are needed to compare performance within and outside of a deceptive context to clarify the effects of deceptive intent.

## Introduction

Deception is an active cognitive process by which the deceiver must inhibit truth-telling while generating false information (Mitchell, 1986). Due to its negative social consequences, there has long been a keen interest in an objective method of detecting deception in the fields of law and security, given for example the inaccuracy of juries and judges in assessing veracity (Appelbaum, 2007). Such objective measures could also aid in the understanding and treatment of psychiatric disorders in which the ability to deceive is impaired (e.g., autism: Sodian and Frith, 1992) or is a symptomatic component (e.g., antisocial personality disorders: Ford et al., 1988). In the early 20th century, the polygraph, relying on peripheral, anxiety-induced autonomic indicators, was proposed as a tool to study lie detection (Larson and Haney, 1932). However, the ability of individuals to defeat these methods by learning to manipulate physiological measures such as skin conductance and heart rate (Honts et al., 1985, 1996), as well as their intrinsic variability (Saxe et al., 1985), has suggested that an objective technology of deception detection requires a greater understanding of the brain processes underlying deception itself. Thus, attention has since turned toward direct measures of brain activity involved with deception utilizing electrophysiological and functional magnetic resonance imaging (fMRI) techniques.

Research on deception using electrophysiology dates back three decades and indicates that scalp electrical potential measures are sensitive to deceptive contexts. There has been a great deal of work focusing on the relationship of deception and event-related potentials (ERPs) with a later onset latency, especially P300s (e.g., Farwell and Donchin, 1991; Johnson et al., 2005). The literature suggests that deceiving requires a higher cognitive workload than truth-telling and that this difference is reflected by changes in the magnitude and latency of specific ERPs (Czigler et al., 2002). However, ERPs with latencies much earlier than P300s are also influenced by deception manipulations, suggesting that deception can influence processing in earlier stages as well. For example, for tasks using visual stimuli, a negative component appearing around 80–180 ms post-stimulus onset, the N1, with greatest amplitudes in the occipital area, is more negative when deception is required, probably due to greater use of early attentive processes (Hu et al., 2011). From about 180–325 ms post-stimulus, an N2 waveform is prominent in frontal and central regions and its change found with deception has been proposed to reflect the mental task of categorizing a stimulus to be lied about and preparing that response (Wu et al., 2009; Hu et al., 2011, Leng et al., 2019).

Neuroimaging has also been shown to be sensitive to experimental manipulation of deception, with studies finding regional activation differences when subjects are practicing deception vs. truth-telling. A recent meta-analysis (Delgado-Herrera et al., 2021) demonstrated substantial involvement of the fronto-parietal network in deception (see also Christ et al., 2009; Lisofsky et al., 2014; Yu et al., 2019), with frontal activations associated with executive functions required to deceive, such as working memory, inhibition, and task switching (Christ et al., 2009); and parietal activations linked to the recruitment of additional resources such as socio-cognitive processes when the task involves social or virtual interactions (Lisofsky et al., 2014); or additional attentional resources when instances requiring deception arise (Christ et al., 2009). However, the ability to use neuroimaging for the detection of deception is hampered by the sheer number and complexity of processes involved in deception such as the cognitive and emotional processes necessary to generate the rationale, intent, and strategies for deception within a given context, as well as those needed to execute a response which is incompatible with the truth (Johnson et al., 2003). Acts of deliberation over deception include weighing risks and benefits, the mind of the other(s) to be lied to, the content and context of the lie, and the recognition of the truth and its inhibition, all governed by many overlapping cognitive processes most likely having a great degree of individual variability (Keckler, 2005). Even the most general taxonomy of the processes involved in deception is complex, grouping them under four sets of cognitive resources: information management, risk management, impression management, and reputation management (Sip et al., 2008). This processing complexity, and the concomitant complexity of its neural underpinnings, has been acknowledged (e.g., Nuñez et al., 2005), and some studies have attempted to differentiate component executive processes used in the deceptive act with manipulations of working memory load (Ganis et al., 2003) or memory content (Nuñez et al., 2005). Nevertheless, the correlative nature of imaging studies, especially when several neural processes are involved, has made interpretation difficult.

Several groups have attempted to use non-invasive brain stimulation such as transcranial magnetic stimulation (TMS) and transcranial direct current stimulation (tDCS) as a more direct approach to detection of deception than imaging. By using results found in electrophysiological and imaging studies to target cortical regions involved with specific aspects of deception, stimulation holds the attractive potential to directly interfere with brain processes involved with producing a deceptive response to produce a measurable difference in performance when being truthful or deceptive (Luber et al., 2009). TMS and tDCS have already been shown to affect behavioral performance in deceptive contexts. Two early studies applied TMS over the motor cortex and found greater cortico-spinal excitability while subjects responded with lies compared to truth (Lo et al., 2003; Kelly et al., 2009). Five studies focused on stimulation of prefrontal cortex (PFC), and while one of them did not show any differences between truth conditions caused by TMS (Verschuere et al., 2012), a series of four other experiments conducted by the same group demonstrated significant TMS effects on deception processes (Karton and Bachmann, 2011, 2017; Karton et al., 2014a,b). Indeed, using both online and offline TMS, Karton and associates found hemispheric differences between truth and lie conditions, with a lower number of deceptive responses with stimulation to left PFC compared to right, as well as an abolishment of the difference seen between truth conditions in the electrical P300 evoked response.

There have also been several studies of deception using tDCS. Two studies stimulated the right temporo-parietal junction and found decreased deceptive responding in a social context (Tang et al., 2017; Noguchi and Oizumi, 2018), while most have focused on PFC stimulation (Priori et al., 2008; Karim et al., 2010; Mameli et al., 2010; Fecteau et al., 2013; Maréchal et al., 2017; Sánchez et al., 2020). Priori et al. found bilateral stimulation increased reaction time in deceitful responses compared to truth, while Mameli et al., Karim et al., and Fecteau et al. found faster RT in lie conditions. Marechal et al. found tDCS to right DLPFC decreased the number of lie responses, and Sanchez et al. found right ventrolateral PFC tDCS disrupted truth-telling, with no effects on lies. Overall, while these TMS and tDCS studies vary in their specific findings, they do indicate that brain stimulation can be effective in producing behavioral differences which depend on deceptive intent. However, most of these studies used offline stimulation—i.e., they evaluated performance changes before and after stimulation. While the offline approach provides important information about the role of specific brain regions in deception, results may be contaminated by the cumulative effects of stimulation that can spread to other brain regions transsynaptically (Beynel et al., 2020). Moving toward a paradigm of direct, online stimulation which can affect behavioral performance on a trial-by-trial basis in specific contexts could afford a means of disrupting deceptive processes on an individual basis with temporal and spatial precision. In addition, these cited studies used trains of rTMS or continuous tDCS which, while effective, cannot provide precise temporal information of the neural mechanisms involved in deception. Single pulses of TMS provide a much more fine-grained time-resolution and allow the experimenter to precisely dissect network activity in time as well as space. This approach was first used by Amassian et al. (1989) to disrupt letter identification, and has successfully been used by our group and others to disrupt complex object recognition in higher visual areas (Luber et al., 2020), self-related episodic memory and self-judgments (Lou et al., 2004, 2010; Luber et al., 2012), numerical cognition (Garcia-Sanz et al., 2022) or cognitive functions assessed via the stop signal task (Bashir et al., 2020), suggesting that single pulse TMS can provide important information regarding the chronometry of complex cognitive functions. The present study attempted to target processes involved with the execution of deceptive responses in a simplified behavioral context as a proof-of-concept for this paradigm.

Disrupting deception with non-invasive brain stimulation, however, is not straightforward. There is no “deception region” of cortex, no “deception network.” Correspondingly, cognitively there is no process central to deception. Deception describes a family of behaviors, all intended to instill a false belief in another person's mind. A particular deceptive action chosen from this family of behaviors is generated from a set of general cognitive processes (e.g., risk processing, Theory of Mind, attention, working memory, etc.). Therefore, studying deception using TMS involves the careful dissection of cognitive processes called on within a deceptive context, which can only be done over a series of experiments. In our preliminary experiment, to focus the application of TMS on the output stages of a deceptive act, we chose a validated deception task, the Guilty Knowledge task (GKT) (Lykken, 1960; MacLaren, 2001). The GKT, in its original form, posed questions concerning a “crime scene” with multiple answer options. The correct answers involved details that only the “criminal” would know. The examiner used physiological indicators during the GKT to look for differences in responses to true and false alternatives (Lykken, 1960; MacLaren, 2001). A simplified analogous playing card version of the GKT was developed for imaging studies using a computer monitor instead of a human examiner (e.g., Langleben et al., 2002). In this type of GKT, subjects are given playing cards, divided into those the subject is instructed to tell the truth about and those they are instructed to deny having. This version of the GKT is arguably the most simplified model of the act of deception. We expected the playing card GKT to minimize the deliberative aspects of deception related to cognitive and emotional processes used in generating a lie since the experimenter controlled what to lie about and when to lie. As Sip et al. (2008) observed, the greatest advantage of using the GKT is that it does not address deception in its totality, but only focuses on a limited set of processes, primarily those involving response selection and inhibition: “if deception is a goal, the most basic scenario requires inhibition of prepotent truth responses to make others believe what we want them to believe,” which is the focus of the GKT. TMS was applied to dorsolateral prefrontal cortex (DLPFC) and medial parietal cortex, two nodes of the fronto-parietal network (FPN) involved with executive processing of the type used in the GKT. The two most important roles of the executive system in the present task were to select the response category (lie/truth), and to inhibit the prepotent truth response related to the lie category. We expected the DLPFC to be involved primarily with truthful response inhibition, and the medial parietal cortex to be involved with response selection given its large role in the mapping of salient stimuli to the proper response category. Both have been shown to be activated in imaging studies of deception (DLPFC: Spence et al., 2001; Lee et al., 2002; Ganis et al., 2003; Kozel et al., 2004; Nuñez et al., 2005; Phan et al., 2005; Feredoes et al., 2011; Ito et al., 2011; medial parietal cortex: Lee et al., 2002; Ganis et al., 2003; Langleben et al., 2005; Mohamed et al., 2006; Sip et al., 2010; Hu et al., 2011; Ito et al., 2011). Executive functions relevant to the GKT such as working memory have long been shown to be affected by TMS to DLPFC (Pascual-Leone et al., 1994), including situations in which the task involved handling of relevant and irrelevant stimuli (Feredoes et al., 2011). The medial parietal cortex was chosen as a target over lateral parietal cortex given that TMS to medial parietal cortex has been shown to modulate executive processing (Lou et al., 2004; Luber et al., 2007); working memory, especially in cases where the number of items to be remembered was high (Luber et al., 2007, 2008); and to disrupt selection of salient stimuli (Mevorach et al., 2006). Past imaging work found a strong network node in midline parietal cortex when subjects used working memory to manipulate items in memory, as opposed to just maintaining them over a delay period (Davis et al., 2018). This involvement of midline parietal cortex during item manipulation, high-capacity item maintenance, and categorization suggested this region's involvement in processing related to the GKT task used here.

The high temporal resolution of TMS also allows not just spatial targeting of deceptive processes of selection and inhibition, but temporal targeting as well. We were able to test whether single pulse TMS, applied at various latencies in relation to onset of test playing cards (0, 80, 160, 240, and 480 ms after stimulus onset), reduced performance during the accuracy of deceptive responses. We based our range of pulse times on the N2 complex of ERP components of visual response, which are observed over a range 150–350 ms after stimulus onset, and whose elements associated with executive processing in the FPN peak between 200 and 300 ms (Folstein and van Petten, 2008; Pires et al., 2014). We expected only the pulses in the middle of this range of times (240 and possibly 160 ms) to disrupt performance in deception conditions.

## Methods

### Subjects

Fifteen healthy subjects (8 females) with a mean age of 30.5 ± 6.7 (SD) years were recruited and signed written informed consent to participate in this 3-day study, approved by the New York State Psychiatric Institute IRB. Seven subjects were Caucasian, three were African American, three were Hispanic, and two were Asian. Subjects were required to have normal or corrected-to-normal vision. All subjects were screened with psychiatric, physical, and neurological examinations, urine drug screens, and pregnancy tests for women of childbearing capacity. Potential subjects were excluded if they had a history of current or past Axis I psychiatric disorders (including substance abuse/dependence) as determined by the Structured Clinical Interview for DSM-IV Axis I Disorders (SCID-NP), a history of neurological disease, or seizure risk factors. The SCID for Axis II personality disorders was also administered, and any potential subject with a history of antisocial personality disorder was excluded.

### Guilty Knowledge Task

Deception studies using GKTs require subjects to answer a series of yes/no questions about stimuli with instructions to answer some questions truthfully and other questions untruthfully (Lykken, 1960; MacLaren, 2001). In a playing card GKT (e.g., Langleben et al., 2002, 2005), subjects are “dealt” a hand and are then shown a playing card on a monitor, along with the question, “Do you have this card?”. They are to respond “no” to designated cards in their hands (those that were to be lied about) and to respond truthfully with a “yes” response to the other cards in their hand or to other cards in the deck. In our design, the GKT is repeated over three sessions and subjects are asked to perform six blocks of 60 trials each in each session (see task procedure). The subjects were “dealt” six cards: three to be lied about and three to be responded to truthfully, and 34 “other” cards not in hand (i.e., all non-face cards in an ordinary deck of playing cards were used). Beyond the large number of card stimuli used, the identity of the six cards in hand was changed every block of trials. This continual change in the identity of the lie and truth cards prevented subjects from learning automatic responses based on a constant stimulus-response mapping, and instead forced them to continue to use the executive processes used by the FPN. By using this variable mapping procedure (e.g., Shiffrin and Schneider, 1977), we expected to keep controlled processing in play: executive processes to continually maintain Lie and Truth categories. Similar information management processes must be used in everyday deceptive behavior, when one must remember what was said to whom while weighing what truth or lies will be told (Sip et al., 2008). The variable mapping procedure also mimicked what happens in a card game, where the cards that might be lied about fluctuate with each new hand.

We also attempted to increase the difficulty for control processes to maintain the Lie and Truth categories by preceding each “Deception” block of trials in which the subject was to attempt to deceive the computer or to say the truth, by an “All-Truth” block using the same hand of cards in both blocks, in which subjects were asked to always respond truthfully (i.e., respond “yes” if the displayed card was in hand, and “no” if it was not). It was expected that in the deception block, in a “Lie” trial, subjects would need to inhibit a more prepotent truthful “yes” response temporarily established by stimulus-response mappings generated in the previous block, thus making the control process involved more vulnerable to TMS disruption.

Further, we attempted to maintain a personal context of being deceptive on the part of the subjects by creating a virtual “Other” they would be deceiving. Subjects were told that the computer would use their responses during a block of trials to guess which cards they had in their hand and that the computer's guess would be displayed at the end of the block. They were told that this guessing program was a work in progress, that they were there to test it by actively trying to fool the computer by lying about some of their cards, and that they succeeded if the computer's guess was wrong. This manipulation was performed to increase subject's incentive to deceive convincingly throughout a session. Moreover, such continued virtual interactions have been shown to elicit strong parietal activations in imaging studies (Lisofsky et al., 2014), leading to an expectation that parietal stimulation might affect the processing associated with that interaction and virtual interaction processes that would elicit stronger parietal activations. The order of card presentation in a block of trials was designed to reinforce the perception that the computer was gradually homing in on the cards in the subject's hand by presenting the subject's cards more and more frequently over the course of the block. One indication that this had been effective came during subject debriefing after their last session. All subjects were surprised to find out that the computer was not trying to guess their cards, and that their efforts to fool it were unnecessary.

### Task Procedure

Subjects were seated in a cushioned chair in the middle of the testing room, facing a computer monitor 100 cm away, with their heads resting on a chin rest. In each session, subjects were asked to perform six blocks of 60 trials. In each block, they were dealt a “hand” of six physical playing cards, displayed along the bottom of the monitor to allow for continuous viewing of the cards throughout the trial. Cards were chosen randomly by a computer before the session, with the only constraint being that the hand contained a mixture of suits.

**Figure 1:**
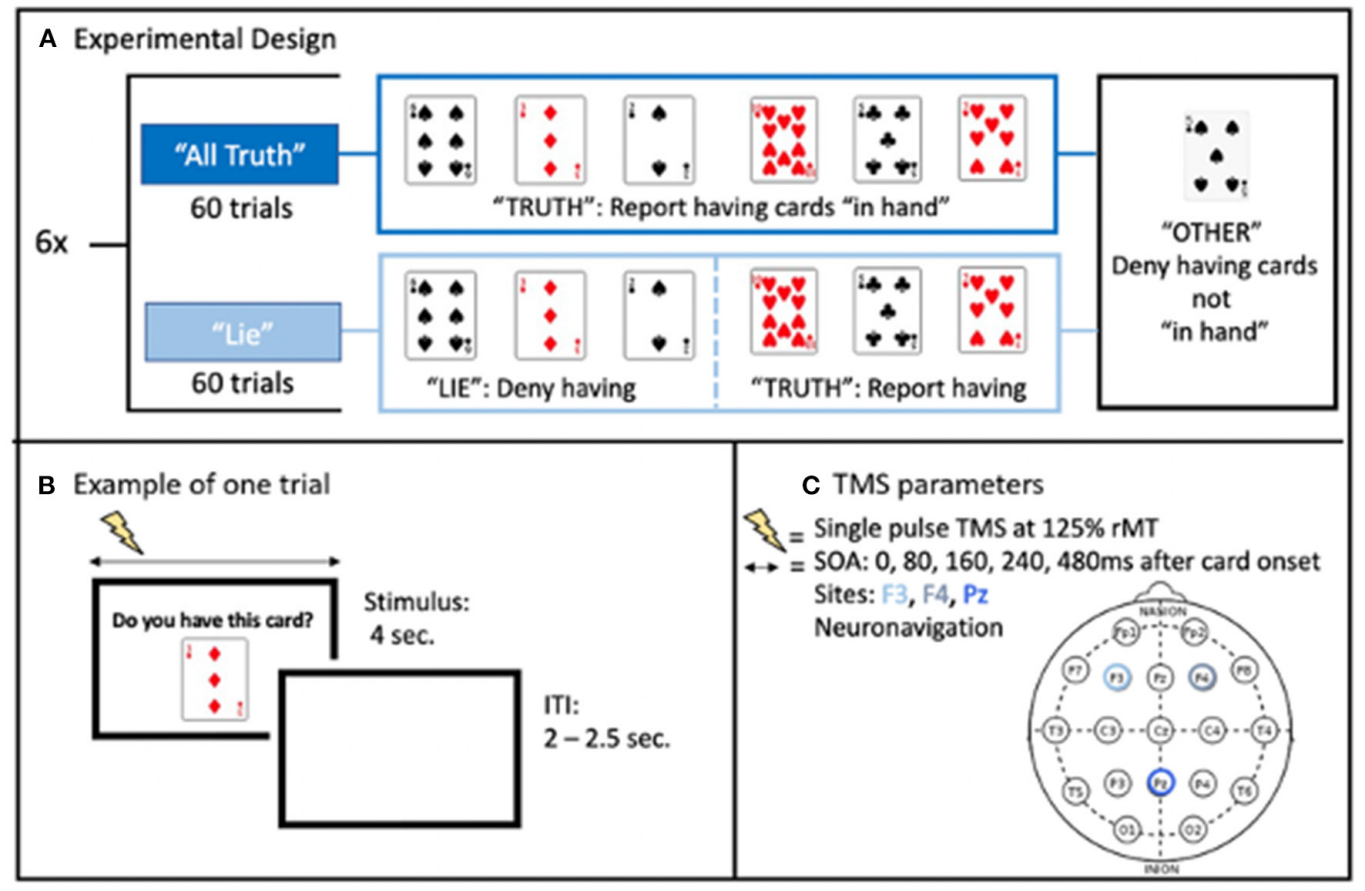
**(A)** Illustration of the experimental design. **(B)** Example of one trial with each card presented for 4 s, during which single pulse TMS was applied, and separated from each other by a random inter-trial interval. **(C)** Stimulation parameters with randomized stimulus onset asynchrony (SOA) relative to the card presentation onset.

The blocks alternated between “All-Truth” blocks, in which subject had to answer truthfully to all trials; and “Deception” blocks, in which they had to either: deny having three of the cards (“Lie” cards: 20 trials), answer truthfully about three others (“Truth” cards: 20 trials), or answer truthfully about not-in-hand cards (“Other” cards: 20 trials) (Figure 1A). Before this second block, they were told that the computer would use their responses to guess which cards they had in their hand. The computer's “guess” of the subject's hand appeared at the end of the block of trials. The trial type for each trial was randomly chosen with two constraints: first, that there were twenty of each of the three trial types over the 60-trial block, and second, that as trial number increased, the probability of an “Other” trial decreased. For a given trial, a number between 1 and 60 was randomly chosen by the computer. If the number was less than the trial number + 6, the card would be chosen from the “in hand” cards (+6 was arrived at empirically to lead to more in hand cards earlier in the block). This resulted in an increased frequency of “in hand” cards over the block of trials. On each trial, a digital image of a playing card was displayed on the monitor for 4 s, with a randomized 2.0–2.5 s interval between displays (Figure 1B). Only numbered cards and aces were used (a forty card “deck”). The display of a card was the cue to respond as to whether it was in their hand or not. Subjects were instructed to confirm or deny their possession of a given card by making a speeded response by button press.

### TMS Application

Single pulse TMS were applied using a figure 8 coil (9 cm diameter) powered by a Magstim 200 stimulator (Magstim Co., Whitland, Southwest Wales, UK). Stimulation intensity was set at 125% of resting motor threshold of the left hemisphere. Resting motor threshold was defined as the lowest intensity needed to evoke motor potentials of at least 50 μV recorded via EMG from the right first dorsal interosseus muscle (FDI) in at least 5 out of 10 stimulations (Rossini et al., 1994). Stimulation was applied over the left DLPFC, right DLPFC, and medial parietal cortex on three different days, with the order counterbalanced across subjects. These three areas are associated with deception (see Introduction) and were targeted using the International 10–20 EEG system (F3, F4, Pz, respectively). Monophasic TMS pulses were used, with the TMS-induced electric field going in the posterior-anterior direction. Without electric field modeling or participant's anatomical MRI to serve as a guide, the coil was positioned perpendicularly to the midline for the frontal targets with the handle pointing down for the left and the right DLPFC. For the medial parietal cortex, the coil handle was parallel to the midline and pointing downward. The coil was positioned and continuously monitored during each session using a computerized frameless stereotaxic system (Brainsight, Rogue Research, Montreal, Canada) based upon a standard brain (MNI). In each trial, single pulse TMS was delivered with a stimulus onset asynchrony (SOA) between card presentation and the TMS pulse of either 0 (Control condition), 80, 160, 240, or 480 ms (Figure 1C). The SOA in each trial was randomized, with the constraint that there were four trials of each SOAs for each of the three trial types (truth, lie, other) in each 60-trial block. This resulted in 24 trials per SOA per trial type over a given scalp location. There were three sessions per subject, each session lasted ~3 h. At the end of the third session, the subject was debriefed as to the purpose of the study and the real nature of the stimulus presentation program.

Performance was assessed by measuring response accuracy and reaction time (RT), as well as a score that combines accuracy and RT into a composite score called the inverse efficiency score (IES = RT/Accuracy: Townsend and Ashby, 1978). We included the IES as it is a robust predictor in detecting deception from truth telling (Monaro et al., 2021). We expected single pulse TMS to disrupt deception processes specific to executive control of stimulus/response selection and inhibition, as reflected by lower accuracy, slower RT and/or higher IES, only for “Lie” cards for stimulation applied over the parietal cortex and the DLPFC; and only for stimulation applied at 240 ms (and possibly 160 ms), when these specific processes are critically active, as reflected by the peak activity of N2 ERP components. We did not expect any changes for stimulation applied at 0 ms, when these executive processes had not yet been called into play. While the “extra” processing required to select a deceptive response was expected to make performance in the Lie category vulnerable to TMS, we did not expect any changes in reaction time, accuracy, or IES when “truth” or “other” cards are presented, as response selection can occur according to the truthful well-learned default.

### Analysis

Omnibus repeated measure ANOVAs were run for median reaction time (RT), mean accuracy (% correct), and inverse efficiency score (IES) calculated as the ratio between RT and accuracy. The All-Truth Blocks were not included in the analysis since they were only used as a primer to make the inhibition of truthful answers more challenging in the subsequent Deception Blocks. Analyses were performed only on the Deception Blocks with the following within-subjects factors: Site (Left DLPFC, Right DLPFC and medial parietal), Card Conditions (Truth, Lie, and Other), and SOA (0, 80, 160, 240, 480 ms).

## Results

Fifteen subjects were enrolled. Data from one subject was excluded due to excessively long reaction times which were greater than two standard deviations above the group mean in all conditions (our *a priori* defined criterion for drop-out). All data are reported as mean ± standard deviation.

### Accuracy Performance in the GKT Task

There were no effects on performance accuracy caused by TMS or by deception. The repeated measures ANOVA revealed a main effect of Card Condition [*F*_(2, 26)_ = 3.85, *p* = 0.03, η^2^ = 0.031] on accuracy. *Post-hoc* Bonferroni comparisons showed that, while no differences were found between “Lie” (95.18 ± 5.99%) and “Other” cards (97.43 ± 4.14%) [*t*_(13)_ = −2.05, *p* = 0.15] or between “Lie” and “Truth” cards (94.52 ± 5.91%), [*t*_(13)_ = 0.59, *p* > 0.05], a significant difference was found between “Truth” cards and “Other” cards (97.43 ± 4.14%) [*t*_(13)_ = 2.64, *p* = 0.04], suggesting that participants were more accurate when responding truthfully about the cards that were not in their hand compared to the cards that were. Results also revealed a main effect of SOA [*F*_(4, 52)_ = 3.20, *p* = 0.02, η^2^ = 0.003] but Bonferroni corrections did not reveal any significant differences between each pairwise comparisons (*p* > 0.05 for all, see Table 1). There was no main effect of Site [*F*_(2, 26)_ = 0.686, *p* = 0.51] and no interaction was found between Site and Card Condition, Site and SOA, or Card Type and SOA (*F* < 1 for the three interactions), nor was the two-way interaction between the three factors significant [*F*_(16, 208)_ = 1.25, *p* = 0.23].

**Table 1:** Mean percent accuracy and reaction time (in seconds) and their standard deviation for each SOA, card condition, and site.

**SOA**	**0 ms**	**80 ms**	**160 ms**	**240 ms**	**480 ms**
Accuracy	95.38 (5.28)	95.37 (5.30)	95.92 (4.97)	95.47 (4.77)	96.42 (4.29)
Reaction time	1.00 (0.21)	1.03 (0.22)	1.03 (0.23)	1.04 (0.24)	1.04 (0.20)
**Card condition**	**Lie**	**Other**	**Truth**		
Accuracy	95.18 (5.99)	97.43 (4.14)	94.52 (5.91)		
Reaction time	1.04 (0.24)	1.01 (0.22)	1.03 (0.20)		
**Site**	**Left**	**Right**	**Parietal**		
Accuracy	96.51 (4.40)	96.23 (6.89)	94.37 (7.69)		
Reaction time	1.06 (0.31)	1.03 (0.25)	1.00 (0.19)		

### Reaction Time Performance in the GKT Task

**Figure 2:**
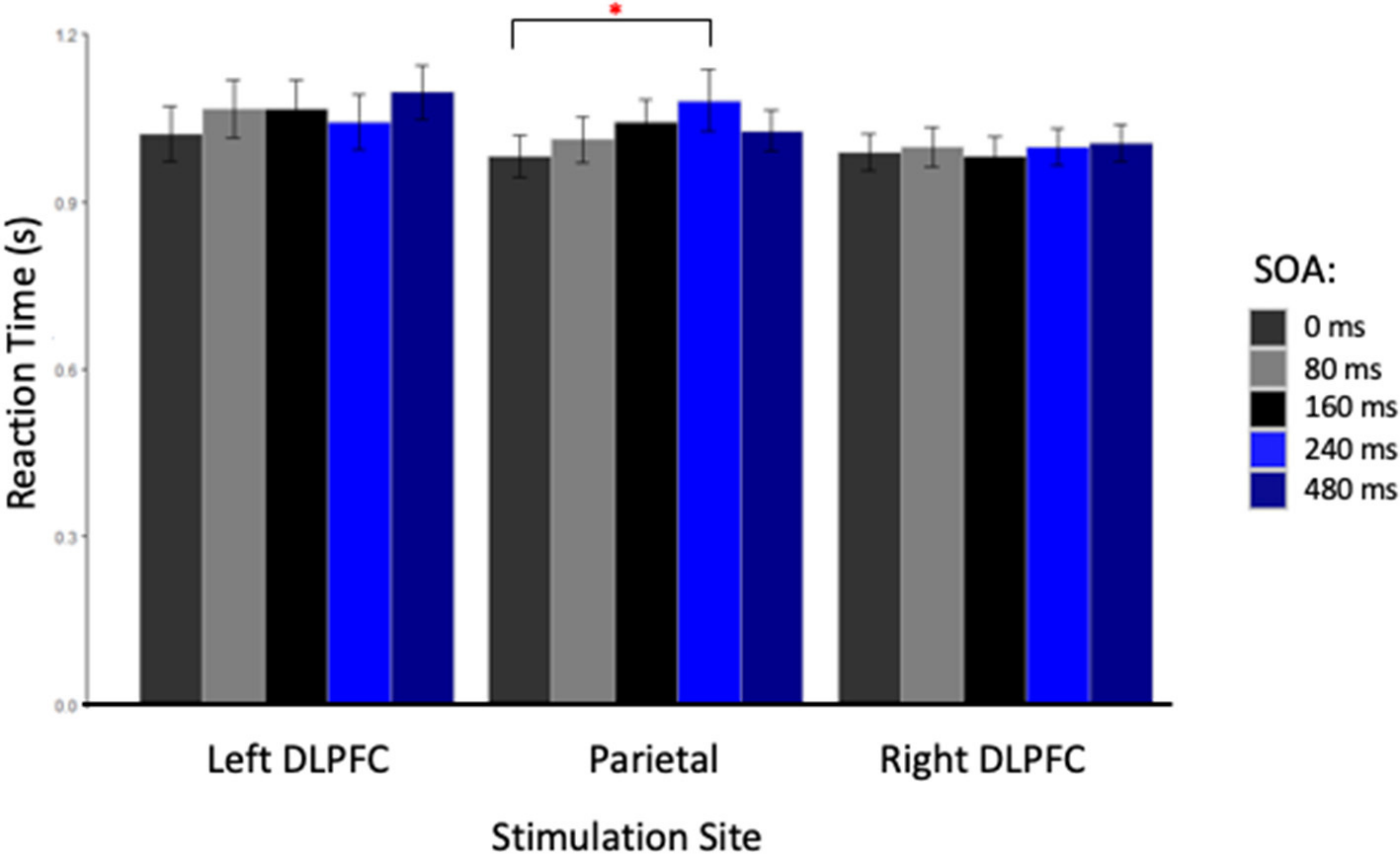
Reaction Time in seconds for each stimulation site at each stimulus onset asynchrony (SOA). The red star indicated a significant difference between reaction times for TMS applied at 0 and 240 ms, only when the parietal cortex was stimulated.

The repeated measures ANOVA did not reveal a main effect of Card Condition [*F*_(2, 26)_ = 1.27, *p* = 0.30], Site [*F*_(2, 26)_ = 0.49, *p* = 0.62], or SOA [*F*_(4, 52)_ = 2.45, *p* = 0.06] (see Table 1). However, a significant interaction was found between Site and SOA [*F*_(8, 104)_ = 2.26, *p* = 0.03, η^2^ = 0.010], suggesting that TMS had location and latency specific effects on RT. *Post-hoc* Bonferroni comparisons, performed to decompose this interaction revealed a significant difference between stimulation applied at 240 ms SOA at the parietal site (1,080 ± 330 ms) compared to TMS applied at 0 ms SOA (980 ± 240 ms) [*t*_(13)_ = 3.97, *p* = 0.01] (see Figure 2). No other comparison reached statistical difference threshold. This suggests that applying TMS at 240 ms after stimulus onset slowed participants' performance compared to our control condition. Another interaction was found between Stimulation Site and Card Type [*F*_(4, 52)_ = 3.05, *p* = 0.03, η^2^ = 0.013], and Bonferroni corrected *post-hoc* comparisons revealed a trend toward significance between parietal stimulation when subjects were instructed to lie (1,080 ± 330 ms) vs. when responding to “Other” cards (990 ± 0.210 ms) [*t*_(13)_ = 3.20, *p* = 0.07]. Subjects tended to take longer to lie than to respond neutrally when the parietal region was stimulated.

### Inverse Efficiency Score in the GKT Task

**Figure 3:**
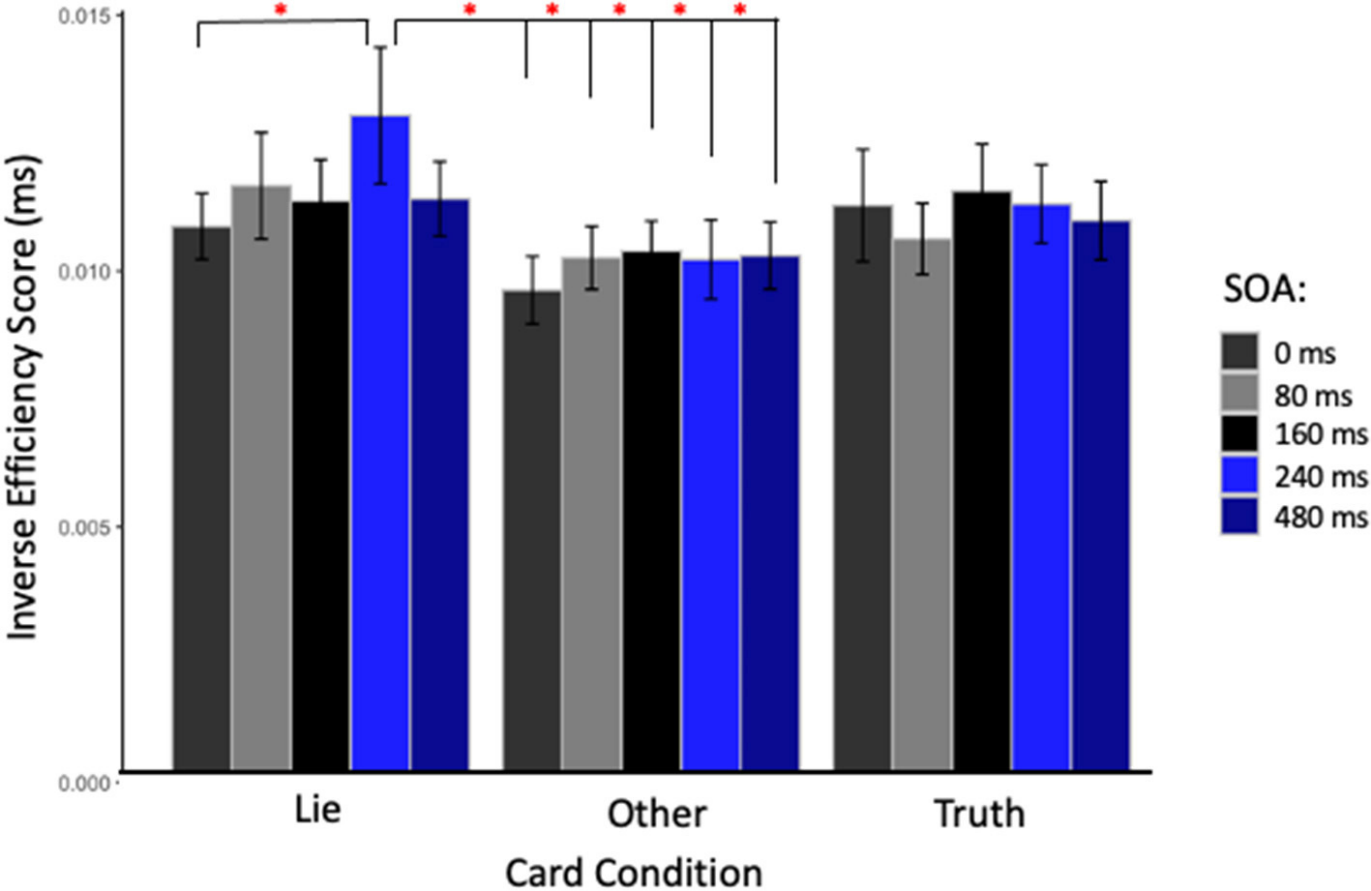
Inverse efficiency score (IES) in milliseconds for TMS applied over the parietal cortex at each SOA and for each card type. Red stars indicate significant IES difference when stimulation was applied at 240 ms after the stimulus onset and subjects were asked to lie, compared to when stimulation was applied at 0 ms in the same condition; or to any other timing when subjects were asked to being truthful about cards not in hand.

The repeated measures ANOVA performed on the IES, a composite measure that integrates reaction time and accuracy and to be a good indicator of deception, found no main effect of Site [*F*_(2, 26)_ = 0.39, *p* = 0.68], SOA [*F*_(4, 52)_ = 1.64, *p* = 0.18], or Card Type [*F*_(2, 26)_ = 2.78, *p* = 0.08]. The interactions between Card Type and SOA, and Site and SOA were not significant [*F*_(8, 104)_ = 0.84, *p* = 0.57; and *F*_(8, 104)_ = 1.52, *p* = 0.16, respectively]. However, the interaction between Card Type and Site was significant [*F*_(4, 52)_ = 2.67, *p* = 0.04, η^2^ = 0.019]. *Post-hoc* Bonferroni-corrected *t*-tests showed that only when the parietal cortex was stimulated, participants were less efficient in responding with a lie about the cards in their hands than with the truth about the cards that they did not have in their hands (“Lie” cards = 0.011 ± 0.003 ms vs. “Other” cards = 0.010 ± 0.003 ms, *p* = 0.02). Finally, the two-way interaction between the three factors was close to significance [*F*_(16, 208)_ = 1.63, *p* = 0.06, η^2^ = 0.021], and the decomposition of this interaction with Bonferroni correction showed that applying TMS over the parietal cortex at 240 ms made participants significantly less efficient when asked to lie (0.013 ± 0.005 ms) than when stimulation was applied at 0 ms (0.011 ± 0.002 ms, *p* = 0.05), mirroring the effect seen with RT alone. Moreover, participants were less efficient at lying when TMS was applied at 240 ms compared to responding with the truth about “Other” cards at every SOA (0 ms = 0.009 ± 0.002 ms, *p* < 0.01; 80 ms = 0.010 ± 0.002 ms, *p* = 0.012; 160 ms = 0.010 ± 0.002 ms, *p* = 0.03; 240 ms = 0.010 ± 0.003 ms, *p* = 0.02; and 480 ms = 0.010 ± 0.002 ms, *p* = 0.016, see Figure 3). In contrast with these TMS effects related to the deception condition found with parietal stimulation, no differences were found in IES with TMS applied over right or left DLPFC.

**Figure 4:**
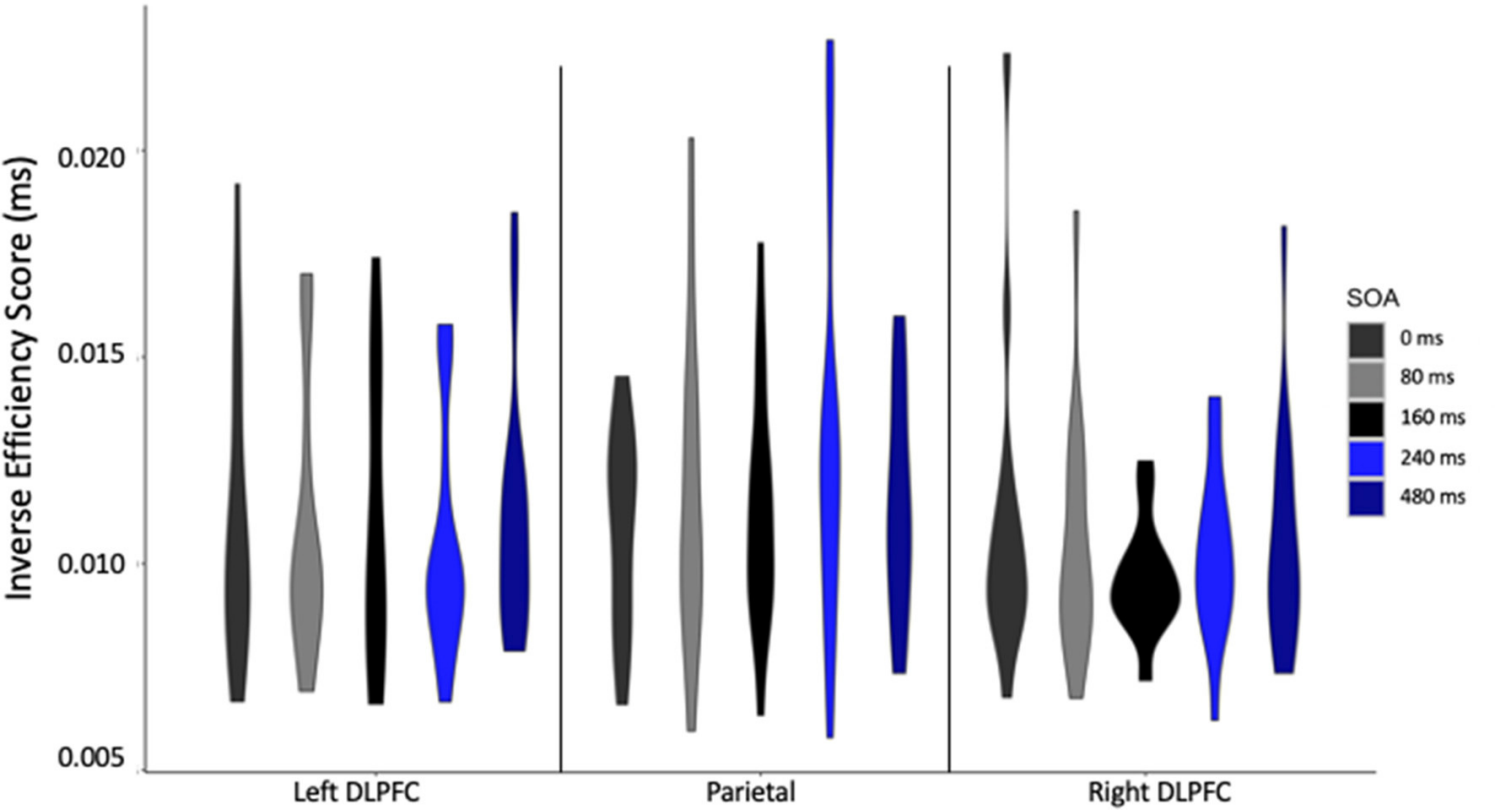
Inverse efficiency score (IES) in milliseconds for each stimulation site, at each stimulus onset asynchrony (SOA) for Lie cards only.

When considering the effect of TMS on IES at the individual level, and focusing on the Lie condition, where the TMS effects were found, some influential interindividual variability can be seen, with some participants less efficient than others (Figure 4). However, this is specific to some of our conditions, for example, participants with high IES in parietal cortex stimulation do not display high IES for left DLPFC stimulation, suggesting that stimulation effects are different at the parietal site. Therefore, future studies might want to reproduce this experiment with larger sample size to understand why some participants show stronger TMS effects than others.

## Discussion

In this preliminary study, we tested whether applying single pulse TMS at specific time points over three nodes of the fronto-parietal network while participants performed a playing card guilty knowledge task could disrupt behavioral performance conditional on deception instructions. Our results demonstrated a site- and latency-specific effect of TMS, since performance to “Lie” cards was disrupted only when the parietal cortex was stimulated 240 ms after stimulus onset, therefore supporting a functional role for the midline parietal cortex processes supporting deception.

### Parietal Cortex Involvement in Processes Supporting Deception

The disruption of processes used for deception, as reflected by an increase in reaction time and IES when TMS was applied over the parietal cortex at 240 ms after the stimulus onset, is in keeping with the expanding knowledge of the role of parietal cortex in control of task processing as part of the fronto-parietal executive network. While parietal association cortex has traditionally been associated with sensorimotor control (e.g., grasping with hands, or eye movements toward, salient visual objects; e.g., Rafal, 2006), research over the last few decades has expanded the role of posterior parietal cortex to include more processing preparatory to such actions: identifying objects within a visual scene according to their salience in relation to goals (Buschman and Miller, 2007; Egner et al., 2008) and their affordances (i.e., understanding objects by the actions they afford; Binkofski and Buccino, 2018). Such mapping is done laying out relational inferences between objects and categories structurally in a scene (Summerfield et al., 2020) with object representations formed in relation to their action affordance and task salience but independent of action planning (Kastner et al., 2017). This independence from action allows for the involvement of working memory on object representations (Marois and Todd, 2004; Davis et al., 2018; Papagno, 2018; using TMS Luber et al., 2007), although ultimately all is in the service of visuomotor transformation based on task-related salience and object affordance (Binkofski and Buccino, 2018). In the present study the choice of the GKT removed many of the processes associated with deception such as risk management, impression management, and reputation management (Sip et al., 2008). Instead, it focused on information management processes requiring involvement of the fronto-parietal network to carry out a deceptive response, primarily those involving response selection and inhibition of prepotent truth. In particular, the categorization of Truth and Lie cards by spatial position, the concurrent placement of the test card in the visual field with the cards in hand, and the complexity of the individual stimuli (six cards in hand, thirty-two other cards, all varied across blocks of trials) would be expected to require the mapping-for-action processes the posterior parietal cortex specializes in. Moreover, it has become clear that the dorsal visual processing stream flowing through the parietal cortex (Mishkin and Ungerleider, 1982) is itself divided into multiple processing streams, with a ventro-dorsal pathway that handles more constant object properties, and a more medial dorso-dorsal stream handling more variable visual context (Sakreida et al., 2016). Given the continued variation in card stimuli, it is not surprising that TMS to the medial parietal cortex could disrupt performance in the present study which would be dependent on this latter pathway, although that remains to be tested by comparing TMS to the two paths.

The finding that the performance disruption only occurred in the Lie condition provides evidence that the added requirement to suppress a prepotent truth and respond with a no places an extra processing burden on the parietal visuomotor transformation mechanisms. The timing of the TMS generating the parietal performance disruption at 240 ms post visual stimulus onset also lines up with event-related potential (ERP) findings: namely, the N2B and N2C components, which occur between 200 and 300 ms post stimulus onset, and which are associated with executive processing in the fronto-parietal control network and with task-related stimulus classification in posterior cortex (Folstein and van Petten, 2008; Pires et al., 2014).

Beyond its involvement in goal-related representation, visuomotor transformation, and executive functions, the parietal cortex is also known to be highly involved in theory of mind and social cognition, and this leads to a second possible mechanism behind the effect on performance in a deceptive context with parietal TMS. The location of the stimulating coil was near the precuneus, which is involved in self-processing (Cavanna and Trimble, 2006; in TMS: Lou et al., 2004) and which has been activated in tasks involving deception in imaging studies (Lisofsky et al., 2014). In our modified version of the GKT, a game-playing context was created in which subjects were trying to fool a device that was trying to guess their cards by trying to “read” their responses. At the same time, the computer appeared to be narrowing in on the cards in their hands by querying about them with a greater and greater frequency, although it did not always guess them correctly at the end of a block. According to debriefings, subjects were generally convinced that this back-and-forth game with the computer was real, and that the computer was getting closer and closer to knowing what cards were in their hands, even though they were lying about half of them. Thinking about their active deceptive role while moving progressively closer to being “caught” may have resulted in precuneus self-related processing in our subjects which contributed toward the Lie response, which TMS to this site could disrupt. However, given our current design, it is difficult to dissociate those two cognitive vs. socio-cognitive potential explanations, and will require future studies to better answer this question.

### Lack of Prefrontal Effect on Deceptive Performance

As major nodes in the fronto-parietal network, right and left DLPFC were chosen as TMS sites, given that, as with the parietal cortex, we expected they could be active during the GKT task, where the sorts of controlled processing performed by DLPFC might be needed to execute the deception task. This expectation has been supported by several electrophysiologic, neuroimaging and brain stimulation studies with TMS and tDCS all demonstrating significant involvement of the DLPFC in processing during deception tasks. However, our results failed to show an effect of our deception manipulation. Several factors could have been responsible for this.

First, using the present version of the playing card GKT, DLPFC processing might not be essential to performance, a possibility supported by the present results. It is possible that the parietal part of the FPN network alone was sufficient to perform the task, as the DLPFC tends to be activated with added task complexity (e.g., Feredoes et al., 2011). Given that the GKT used here has arguably the simplest form a deception task can take, with a framework of simple categorization using well-known stimuli, this may well be the case. The addition of a working memory component to the deception task (e.g., Ganis et al., 2003), or a greater number of categories, such as a “yes” lie condition, or a more complicated decision rule about which cards to lie about, could be expected to promote the involvement of DLPFC and give TMS applied there something to disrupt.

Second, the site of TMS application may not have been optimal, both within the DLPFC, or more generally, within prefrontal cortex. Supporting the latter case, some previous imaging studies using GKT found deception-related activations in ventrolateral PFC rather than DLPFC (Langleben et al., 2005; Spence et al., 2008). Future TMS research investigating GKT and deception processing should utilize targeting using individualized fMRI (Beynel et al., 2020)—a limitation of the present study (see below). Along the lines of choice of stimulation site, both imaging and stimulation studies of deception have noted more lateral, and bilateral, prefrontal involvement (e.g., Priori et al., 2008; Sandrini et al., 2008). Interhemispheric compensation could have prevented a TMS effect, especially in response to single pulse TMS. Future studies might be designed to explore this possibility by using bilateral stimulation of the DLPFC concurrently using two stimulation coils (Santarnecchi et al., 2018).

A third potential reason for the lack of a PFC-based disruption could have been the timing of the TMS pulses. The range of SOAs for TMS (0–480 ms) was centered on the time period between 150 and 300 ms, when visual processing involved with task-relevant classification that we hoped to affect primarily occurs, as reflected in the activity (and frontal and posterior distribution) of the N200 complex of ERP components. However, TMS pulsed at later SOAs beyond the range used here may have affected frontal processing also associated with deception, as indicated by later frontally distributed ERP components that have been shown to be involved with deception (e.g., Johnson et al., 2008). Future studies might want to more closely coordinate ERPs, as proxies for the dynamics of processing, with the timing of TMS pulses: for example, using a closed loop TMS approach by sending pulses when changes in ERP magnitude are detected, therefore replicating, and extending Karton and Bachmann (2017).

A fourth potential reason is more general: while a single pulse at 240 ms SOA might work to disrupt the kind of processing occurring in parietal cortex when deception is required, the stimulus parameters used in this study may have not been appropriate to do so in DLPFC. For example, while a single pulse might not be effective, a short train of pulses might be, or single pulses at a higher intensity than used here.

### Lack of Difference in Truth and Lie Performance

An interesting result in this study was the absence of difference between Truth and Lie conditions, in contrast to the observed worsening of performance in the Lie condition compared to the “Other” card condition. It is worthy of consideration that in many deception tasks, RT in lie conditions is observed to be slowed relative to truth conditions (e.g., Seymour et al., 2000; Spence et al., 2001; Ganis et al., 2003), and that this is offered as evidence that the act of deception requires additional, time-consuming executive processing beyond what is required for truthful responses. While this is often the case, there have been other studies in which there was no difference in RT between lie and truth (Kozel et al., 2005; Abe et al., 2006), or in which truth response was actually slower (Langleben et al., 2005). As these studies indicate, RT differences in lie vs. truth conditions are task dependent. Relative increases in the lie condition in some deception tasks may have to do with increased executive processing as responses are produced which conflict with prepotent responses to the truth. Production of a deceptive response in our playing card GKT may have relied less upon these processes, as what is to be lied about has been clearly demarcated well ahead of response production. Here, deception may rely upon keeping lie and truth categories clear in working memory, as a visual search matching the test card and cards in hand proceeds, while this was not the case in responding to “Other” cards.

### Conclusions and Limitations of the Study

While TMS offers a means to interfere with cortical processing associated with deception, there are many challenges due to the large number of processes contributing to deceptive acts, including: deciding who to lie to, when to lie in a given context, and what to lie about, assessing the social consequences of lying, monitoring the success of the lie and keeping track of what was lied about, as well as the immediate processing involved with performing an act of deception, categorizing the perceptual stimuli in the context of the lie and suppressing the default of telling the truth in response to a query. In this study, we deliberately limited the set of processes needed for deceptive performance to the latter group needed for immediate response selection and inhibition, to establish an initial proof-of-concept for this TMS paradigm to explore the underlying neural mechanisms of deception. Single pulse TMS applied to medial parietal cortex at 240 ms after visual stimulus onset significantly slowed response and decreased performance efficiency when stimuli were presented to be lied about, while no effects of TMS on performance were observed with stimuli to be responded to truthfully. This result provides evidence that TMS can be used to target specific processes and network nodes involved with producing deceptive actions in the GKT, and that medial parietal cortex is such a node. However, TMS to DLPFC, a prefrontal region implicated in deception across many imaging, electrophysiological and brain stimulation studies, did not produce any change in deceptive performance in our specific implementation of the GKT. A number of reasons for this lack of frontal effects were suggested, involving the choices of GKT task, target site and method, and TMS timing and other parameters, and a number of future directions for future TMS research were pointed out.

Two other limitations should be pointed out. First, the sample size was relatively small, such that although significant and interpretable effects were found they cannot be generalized, and more subjects would be required to reduce interindividual variability and conduct more powerful and meaningful statistical analyses. Second, the targeting approach represents another limitation since the 10–20 EEG approach was used to target the DLPFC and the parietal cortex. While this method offers easy and cheap technique it has been found to often miss the desired target (e.g., Herwig et al., 2003). Spatial targeting could be improved by first obtaining functional brain images specific to this version of the GKT with fMRI, and then using the individual brain images to guide the selection of TMS targets on an individual subject basis, which has been found to be the most effective TMS targeting approach (Beynel et al., 2020). In addition to allowing for finer positioning of the coil, using anatomical MRI could also increase TMS efficacy with an optimal coil orientation, defined by maximizing the strength of the electric field perpendicularly to the closest sulcus (Janssen et al., 2015). Finally, the use of a playing card GKT provided information on the dynamics and neural substrates necessary for the execution of a simple deceptive response, corresponding most directly to bluffing or deceiving in a card game. Future TMS studies are needed to test whether these results generalize to other tasks in which the substance of the deception is not based on simple, arbitrary categories. A more ecologically valid approach might examine deception using real-world knowledge, both autobiographical and more general. Using more complex knowledge representations could illuminate more prefrontal processes of interference and conflict resolution, response inhibition, and higher-level cognitive control that may be more central to understanding and manipulating real-world deception.

To summarize, we demonstrated that single pulse TMS can interfere with ongoing processes used in a deceptive action, providing spatial and temporal information about the neural activity underlying them, and providing an initial step toward using brain stimulation to work out the complex interplay of neural processing required for deception. The utility of such research is broad and could be developed, for instance, as an objective method of detecting deception in the fields of psychiatry and of law and security. In carrying out our paradigm, we succeeded in what must be the first order of business in any study of deception- maintaining a continuous context of deception for the participants throughout their performance of the task- as supported by the fact that each believed they were working against a computer that was actively trying to guess their cards, and were surprised to find out that this was not so. However, these preliminary results cannot clarify whether the TMS affected processes of category selection and inhibition of prepotent response while they were specifically employed under a deceptive intent, or whether the TMS would have had similar performance effects under different (non-deceptive) intent. This requires further studies manipulating deceptive context, for instance by adding a control condition using the same experimental design in which participants would be asked to inhibit the predominant response for certain cards but without being asked to lie. This would be a next step in a series of future studies using TMS needed to explore the neural basis for deceptive actions by examining the component general processes used, both within and outside of a deceptive context, for which the present study provides an initial first step.

## Data Availability Statement

The datasets presented in this study can be found in online repositories. The names of the repository/repositories and accession number(s) can be found below: Open Science Framework: https://osf.io/u2npy/.

## Ethics Statement

The studies involving human participants were reviewed and approved by New York State Psychiatric Institute IRB. The patients/participants provided their written informed consent to participate in this study.

## Author Contributions

BL and SL: conceptualization, funding acquisition, investigation, methodology, project administration, and supervision. TS, MP, and KT: data curation. BL, LB, and HG: formal analysis. LB and HG: visualization. BL, LB, HG, TS, MP, KT, and SL: writing–review and editing. All authors have read and agreed to the published version of the manuscript.

## Funding

This research was supported by a grant from the Defense Advanced Research Projects Agency (DARPA).

## Author Disclaimer

The opinions expressed in this article are the author's own and do not reflect the views of the National Institutes of Health, the Department of Health and Human Services, or the United States government.

## Conflict of Interest

BL, LB, HG, and SL were supported by the NIMH Intramural Research Program (ZIAMH002955). This work was done while BL and SL were at Columbia University, prior to their NIMH employment. SL is an inventor on a patent on TMS technology assigned to Columbia University. SL has received grant support from the Brain and Behavior Research Foundation, the Stanley Medical Research Foundation, Neosync, Nexstim, NIH, and Brainsway. The remaining authors declare that the research was conducted in the absence of any commercial or financial relationships that could be construed as a potential conflict of interest.

## Publisher's Note

All claims expressed in this article are solely those of the authors and do not necessarily represent those of their affiliated organizations, or those of the publisher, the editors and the reviewers. Any product that may be evaluated in this article, or claim that may be made by its manufacturer, is not guaranteed or endorsed by the publisher.
